# Non–HDL to HDL cholesterol ratio and coronary outcomes in U.S. adults: a cross-sectional and prospective NHANES analysis

**DOI:** 10.1186/s40001-026-03977-x

**Published:** 2026-01-30

**Authors:** Haibin Xu, Baohong Yao

**Affiliations:** 1 Cardiovascular Medicine, First Affiliated Hospital of Huzhou University, Huzhou, 313000 China; 2 General Medicine, First Affiliated Hospital of Huzhou University, Huzhou, 313000 China

**Keywords:** NHANES, Non–HDL/HDL cholesterol ratio, Coronary heart disease, Mortality, Lipid markers

## Abstract

**Background:**

Coronary heart disease (CHD) remains a leading global cause of morbidity and mortality. Dyslipidemia—particularly elevated non-high-density lipoprotein cholesterol (non–HDL-C) and reduced high-density lipoprotein cholesterol (HDL-C)—is a key risk factor for CHD. The ratio of non–HDL-C to HDL-C (NHHR) has been proposed as an integrative marker of lipid-related risk. We separately examined the cross-sectional association of NHHR with CHD status and its prospective association with long-term mortality in a nationally representative U.S. cohort.

**Methods:**

We analyzed data from adults (aged ≥ 18 years) in NHANES 2005–2016. NHHR was calculated as non–HDL-C divided by HDL-C. Cross-sectional associations between NHHR and self-reported CHD were assessed using multivariable logistic regression. Prospective associations of baseline NHHR with all-cause and cardiovascular mortality were examined using Cox proportional hazards models. Restricted cubic splines (3 knots) were used to explore potential nonlinear relationships.

**Results:**

Among ~ 20,000 adults, higher NHHR was paradoxically associated with lower odds of self-reported CHD. In fully adjusted models, participants in the highest NHHR quartile had an odds ratio of 0.39 (95% CI, 0.31–0.50; *p* < 0.0001) compared to the lowest quartile. In longitudinal analyses over a median follow-up of 7–10 years, higher NHHR was associated with lower hazard of all-cause and cardiovascular mortality. Spline analyses suggested a U-shaped relationship, with nadirs at NHHR ≈3.4 for all-cause and ≈3.3 for cardiovascular mortality (*P* for nonlinearity < 0.001).

**Conclusions:**

In this large NHANES-based observational study, NHHR was cross-sectionally associated with lower CHD prevalence and longitudinally associated with reduced mortality. While similar patterns have been reported in prior NHANES studies, our analysis contributes additional insight by jointly modeling both CHD and mortality outcomes using nonlinear spline approaches and subgroup analyses. The paradoxical inverse association with CHD may reflect residual confounding or reverse causality. These findings remain exploratory and require cautious interpretation and further validation.

**Supplementary Information:**

The online version contains supplementary material available at 10.1186/s40001-026-03977-x.

## Introduction

Coronary heart disease (CHD) arises from atherosclerotic narrowing of the coronary arteries, leading to impaired myocardial perfusion. This can manifest as angina, myocardial infarction, or sudden cardiac death. Globally, CHD remains a major contributor to cardiovascular mortality, accounting for a large proportion of the approximately 17 million cardiovascular deaths annually. The burden of CHD has continued to rise due to aging populations and the increasing prevalence of modifiable risk factors such as unhealthy diets, physical inactivity, and tobacco use [1, 2].

Dyslipidemia plays a central role in the pathogenesis of atherosclerosis and CHD. Among lipid measures, non–HDL-C represents the total burden of atherogenic lipoproteins and has been shown to be a stronger marker of cardiovascular risk than low-density lipoprotein cholesterol (LDL-C) alone [3]. HDL-C, conversely, has traditionally been viewed as cardioprotective, although extremely high HDL-C levels may reflect dysfunctional particles [4]. Given the limitations of using individual lipid parameters, the ratio of NHHR has emerged as a composite index that may better reflect the balance between atherogenic and protective lipoproteins.

Recent studies have examined NHHR in two distinct contexts: first, its cross-sectional association with prevalent CHD, and second, its prospective relationship with long-term mortality. In the context of CHD, several studies have observed that individuals with higher NHHR tend to have greater odds of reporting CHD at baseline, even after adjusting for traditional risk factors [5]. Separately, multiple cohorts have reported that elevated NHHR is associated with increased all-cause or cardiovascular mortality risk. Furthermore, several cohort analyses in diverse populations have observed nonlinear relationships between NHHR and mortality outcomes. In a U.S. diabetic and prediabetic cohort, a U-shaped association was noted between NHHR and all-cause mortality (with an L-shaped pattern for cardiovascular mortality) [6]. Among hypertensive adults, NHHR showed a U-shaped relationship with mortality risk, indicating possible threshold effects [7]. In patients with nonalcoholic fatty liver disease, higher NHHR was associated with an increased risk of mortality [8]. Likewise, in critically ill patients with sepsis, NHHR exhibited a nonlinear U-shaped relationship with short-term mortality [9]. Collectively, these findings indicate that NHHR may serve as a valuable risk marker, capturing information beyond conventional lipid measures.

While several recent studies have used NHANES data to explore the relationship between NHHR and either CHD or mortality, most focused on single outcomes or specific subgroups (e.g., diabetic or hypertensive populations), and did not systematically evaluate potential nonlinear associations or subgroup effects. Our study extends this body of work by jointly examining both cross-sectional associations with self-reported CHD and prospective associations with long-term mortality in a large, nationally representative sample of U.S. adults. We further contribute by modeling nonlinear dose–response patterns using restricted cubic splines and by evaluating potential effect modification across demographic and clinical subgroups. These analyses were conducted independently to preserve clarity between cross-sectional and longitudinal frameworks.

## Methods

### Data source and study population

The National Health and Nutrition Examination Survey (NHANES) is a comprehensive program designed to assess the health and nutritional status of adults and children in the United States. It uniquely combines interviews with physical examinations, covering demographic, socioeconomic, dietary, and health-related topics. The survey uses cross-sectional data to represent the entire U.S. population, ensuring broad applicability and relevance. This study solely utilizes publicly available NHANES data, eliminating the need for additional ethical approval. For more details on ethical oversight, refer to the NCHS Ethics Review Board (ERB) Approval: NCHS ERB Approval at (https://www.cdc.gov/nchs/nhanes/irba98.htm).The data can be freely accessed through the CDC's NHANES website: NHANES Data Access at https://wwwn.cdc.gov/nchs/nhanes/Default.aspx.

**Fig. 1:**
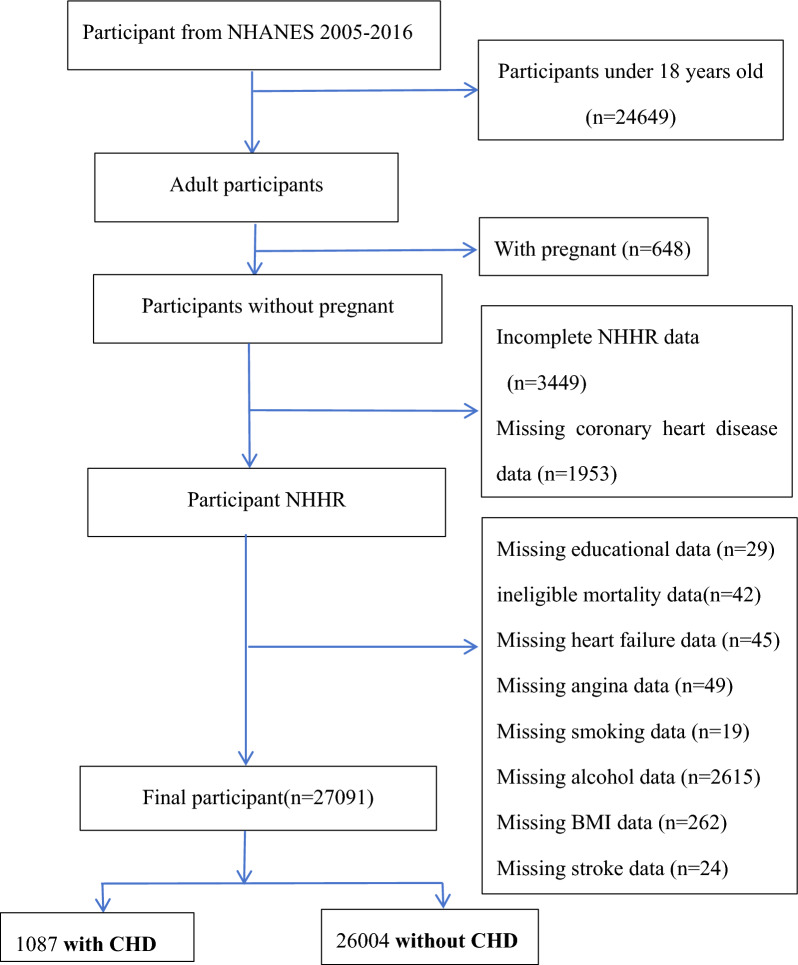
Flowchart of Participant Selection from NHANES 2005–2016

In this study, to evaluate the association between NHHR and the prevalence and mortality of coronary heart disease (CHD), the dataset was collected from NHANES during six cycles (2005–2006, 2007–2008, 2009–2010, 2011–2012, 2013–2014, and 2015–2016). Initially, a total of 60,936 participants were identified across these six cycles. Through a methodological screening process, certain demographic characteristics were excluded as follows: individuals under 18 years old (24,649), pregnant women (684), individuals lacking total cholesterol (TC) or high-density lipoprotein cholesterol (HDL) data (3,449), those who did not provide CHD information (1,953), and those lacking other covariate data. After a systematic exclusion process, 27,091 participants met the inclusion criteria (Fig. 1). Missing values were handled via complete-case analysis. We assessed the proportion and patterns of missingness and found that excluded cases did not significantly differ in baseline characteristics compared to the analytic sample (data not shown).

### Exposure assessment

The focal exposure variable of this study is the NHHR, which is the independent variable in the exposure assessment. It was calculated as the non-high-density lipoprotein cholesterol (non-HDL-C) to high-density lipoprotein cholesterol (HDL-C) [10]. To derive non-HDL-C, HDL-C was subtracted from total cholesterol (TC), and the lipid profiles of fasting individuals were analyzed. Only fasting individuals were included to ensure comparability of lipid measurements and to reduce variability caused by postprandial status. However, this approach may limit the generalizability of the findings to non-fasting populations. An automated biochemical analyzer performed enzymatic tests to assess TC and HDL-C levels. For the determination of TC concentration, the study used the Roche Cobas 6000 and Roche Modular P chemistry analyzers in the analytical procedures. NHHR was divided into weighted quartiles: Q1 (< 1.85), Q2 (1.85–2.45), Q3 (2.46–3.10), and Q4 (> 3.10), based on the survey-weighted population distribution. Quartiles were defined empirically from the weighted distribution of NHANES participants, following the analytic guidelines for continuous biomarkers in complex surveys (NCHS, 2018). NHHR was derived from a single baseline lipid measurement, which may not fully capture long-term lipid status. This limitation is acknowledged in the Discussion.

### Outcome ascertainment

To reflect the distinct nature of our outcomes, we employed separate analytic frameworks: cross-sectional logistic regression to assess associations between NHHR and self-reported CHD at baseline, and prospective Cox proportional hazards models to evaluate the relationship between NHHR and subsequent mortality over follow-up. These analyses were conducted independently to avoid conflating cross-sectional and longitudinal associations under a single predictive model.

The outcomes of interest in this study were coronary heart disease (CHD) and mortality. CHD prevalence was assessed entirely by self-report, based on the standard NHANES questionnaire. Trained interviewers asked participants: “Has a doctor or other health professional ever told you that you had coronary heart disease?” Participants responded “yes” or “no.” If they could not respond or did not know, their responses were coded as missing. CHD prevalence was based on self-reported physician diagnosis using the NHANES questionnaire.

For mortality outcomes, we used the NHANES Public-use Linked Mortality File (https://www.cdc.gov/nchs/data-linkage/mortality-public.htm), which is linked to the National Death Index (NDI) through a probabilistic matching algorithm. Mortality was determined according to the International Classification of Diseases, 10th Revision (ICD-10). Cardiovascular mortality was identified by ICD-10 codes (e.g., I50 for congestive heart failure, cerebrovascular accident/stroke, and others). Follow-up time for each participant was calculated from baseline examination until death or censoring on December 31, 2019. Information on CHD severity, duration, or physician-initiated interventions (e.g., medications, lifestyle counseling) was not uniformly available in NHANES and therefore could not be incorporated.

### Covariates

Covariates that could potentially influence the relationship between NHHR and coronary heart disease (CHD) and mortality were collected through interviews and medical examinations. These included sociodemographic and lifestyle characteristics such as age, gender, race/ethnicity, educational level, marital status, smoking status, drinking status, body mass index (BMI), hypertension, diabetes, total cholesterol (TC), and high-density lipoprotein cholesterol (HDL-C). Demographic data on gender (male, female), age (in years), race (Mexican American, Other Hispanic, Non-Hispanic White, Non-Hispanic Black, Other Races), marital status (Married/Living with partner, Widowed/Divorced/Separated, Never married), educational level (less than high school, high school, more than high school), smoking status (every day, some days, not at all), and drinking status (heavy drinking, moderate drinking) were obtained from interviews. Participants were asked questions such as, "Has a doctor or other health professional ever told you that you have high blood pressure, also called hypertension?", "Has a doctor or health professional ever told you that you have diabetes or sugar diabetes?", "In your lifetime, have you had at least 5 drinks of any alcoholic beverage almost every day for 2 weeks or more?", and "Do you now smoke cigarettes?". Participants responded with a "yes" or "no" answer, and if they refused to respond or were unable to provide an answer, their data were classified as missing. BMI was determined by considering participants' height and weight and categorized into three ranges: 0–25 kg/m^2^ (normal), 25–30 kg/m^2^ (overweight), and > 30 kg/m^2^ (obese). TC and HDL-C levels were measured using an automated biochemical analyzer, with total cholesterol (TC) and HDL-C levels determined using enzymatic methods with the Beckman Synchron LX20. Detailed measurement methods for these variables are publicly available at www.cdc.gov/nchs/nhanes/. Data on lipid-lowering medication use (e.g., statins) were inconsistently available across NHANES cycles and thus were not included in the final adjustment models. As a result, participants using such therapies were retained in all analyses, and their influence may contribute to residual confounding. Data on physical activity, dietary factors, and detailed socioeconomic indicators were not consistently available across all survey cycles; therefore, they could not be included as covariates. Their absence may have introduced residual confounding. Although TC and HDL-C were measured, they were not included as covariates in the final models to avoid over-adjustment bias, as NHHR is derived directly from these components.

## Statistical analysis

To reflect the distinct nature of our outcomes, we employed separate analytic frameworks: cross-sectional logistic regression to assess associations between NHHR and self-reported CHD at baseline, and prospective Cox proportional hazards models to evaluate the relationship between NHHR and subsequent mortality over follow-up. These analyses were conducted independently to avoid conflating cross-sectional and longitudinal associations under a single predictive model.

Analyses were adjusted for available covariates; however, unmeasured historical confounders (e.g., prior lipid levels, long-term treatment history) could not be addressed due to data limitation. Statistical analyses were performed using R software (version 4.3.0). In accordance with NHANES analytic guidelines, survey weights, strata, and primary sampling units were incorporated to account for the complex survey design and to produce nationally representative estimates. Continuous variables were summarized as weighted means with standard errors (SE), and categorical variables were expressed as weighted frequencies and percentages. Group differences were tested using t-tests or Mann–Whitney *U* tests for continuous variables and chi-square tests for categorical variables. Weighted multivariable logistic regression was used to estimate odds ratios (ORs) with 95% confidence intervals (CIs) for the association between NHHR and coronary heart disease (CHD), with quartiles of NHHR analyzed using the lowest quartile as the reference. Cox proportional hazards regression was applied to estimate hazard ratios (HRs) with 95% CIs for all-cause and cardiovascular disease (CVD) mortality, with time-to-event defined as the interval from baseline to death or end of follow-up. Three models were specified for both logistic and Cox analyses: Model 1 was unadjusted. Model 2 was adjusted for age, sex, race/ethnicity, education level, and BMI. Model 3 was further adjusted for smoking status, alcohol use, hypertension, and diabetes. Restricted cubic spline (RCS) models were employed to examine potential nonlinear associations, and subgroup analyses with interaction testing were conducted to assess effect modification across demographic and clinical subgroups. All analyses incorporated NHANES sampling weights, strata and clusters. Odds ratios from survey-weighted logistic regression were reported for CHD prevalence; hazard ratios from survey-weighted Cox models were reported for mortality outcomes. Two-sided *P* < 0.05 was considered statistically significant. Statistical significance was defined as a two-tailed *P* value < 0. 05.

### Restricted cubic spline (RCS) analysis

To explore potential nonlinear associations between NHHR and outcomes, we used restricted cubic spline models. Specifically, splines were constructed with three knots placed at the 5th, 50th, and 95th percentiles of NHHR to capture potential extremes and mid-distribution behavior. Nonlinearity was tested by comparing the spline model to a linear model using a likelihood ratio test. We report two key *P*-values: P-overall (testing for any association between NHHR and outcome) and P-nonlinear (testing whether a nonlinear fit significantly improves model fit over a linear term). These values help determine whether spline modeling is warranted.

## Results

### Characteristics of the study population

Logistic regression and Cox proportional hazards models were selected given the binary and time-to-event outcomes, respectively, consistent with standard epidemiologic practice and prior NHANES literature. Among U.S. adults in NHANES 2005–2016, participants with CHD had lower NHHR than those without CHD (2.84 vs 2.96; *P* = 0.01) and lower TC and HDL-C (both *P* < 0.0001). CHD cases were older (*P* < 0.0001). Distributions differed by race/ethnicity and sex (higher in men; both *P* < 0.0001). Lower educational attainment was more common in CHD (*P* < 0.0001). Diabetes status, smoking, and alcohol use were each associated with CHD (all *P* < 0.0001). Stroke, angina, congestive heart failure, and prior myocardial infarction were markedly more frequent in CHD (all *P* < 0.0001). Across NHHR quartiles, lower quartiles showed higher CHD prevalence (*P* for overall difference = 0.03). CHD prevalence was substantially higher among participants with CVD and hypertension (both *P* < 0.0001). Mortality status also differed by CHD (*P* < 0.0001) (see Table 1).

**Table 1:** General characteristics of participants in NHANES according to CHD status

Variable	Total	CHD	non-CHD	*p*
NHHR	2.96(0.01)	2.84(0.05)	2.96(0.02)	0.01
TC	5.04(0.01)	4.48(0.05)	5.06(0.01)	< 0.0001
HDL	1.38(0.01)	1.26(0.01)	1.38(0.01)	< 0.0001
Age	47.58(0.25)	67.14(0.48)	46.90(0.24)	< 0.0001
Age group				0.75
< 60	27033 (99.83)	1085(99.78)	25948(99.83)	
≥ 60	58(0.17)	2(0.22)	56(0.17)	
BMI	28.93(0.08)	30.17(0.23)	28.89(0.08)	< 0.0001
Ethnicity				< 0.0001
Black	5535(10.45)	143(6.14)	5392(10.59)	
Mexican	4334(8.26)	103(3.58)	4231(8.42)	
Other	5111(11.81)	144(8.90)	4967(11.91)	
White	12111(69.49)	697(81.38)	11414(69.08)	
Sex				< 0.0001
Female	13533(50.62)	344(33.76)	13189(51.20)	
Male	13558(49.38)	743(66.24)	12815(48.80)	
Education				< 0.0001
9-11th grade (Includes 12th grade with no diploma)	3914(10.86)	180(14.01)	3734(10.75)	
College graduate or above	6191(29.31)	205(21.92)	5986(29.56)	
High school graduate/GED or equivalent	6203(22.56)	267(25.86)	5936(22.44)	
Less than 9th grade	2873(5.45)	163(8.53)	2710(5.34)	
Some college or AA degree	7910(31.83)	272(29.68)	7638(31.90)	
Diabetes				< 0.0001
yes	5058(13.95)	488(42.29)	4570(12.97)	
IFG	1181(4.45)	65(6.30)	1116(4.39)	
IGT	1222(4.19)	55(5.52)	1167(4.14)	
no	19630(77.41)	479(45.89)	19151(78.50)	
Smoke				< 0.0001
former	6668(25.09)	492(45.27)	6176(24.40)	
never	14733(54.22)	400(34.98)	14333(54.88)	
now	5690(20.69)	195(19.74)	5495(20.72)	
Alcohol user				< 0.0001
former	4969(15.31)	353(29.97)	4616(14.80)	
heavy	5441(21.15)	81(7.46)	5360(21.62)	
mild	8743(35.38)	410(42.39)	8333(35.14)	
moderate	4029(16.96)	96(9.58)	3933(17.21)	
never	3909(11.21)	147(10.61)	3762(11.23)	
Stroke				< 0.0001
no	26060(97.13)	915(85.29)	25145(97.54)	
yes	1031(2.87)	172(14.71)	859(2.46)	
Angina				< 0.0001
no	26401(97.88)	736(66.53)	25665(98.96)	
yes	690(2.12)	351(33.47)	339(1.04)	
Congestive heart failure				< 0.0001
no	26239(97.68)	741(72.69)	25498(98.54)	
yes	852(2.32)	346(27.31)	506(1.46)	
Heart attack				< 0.0001
no	25949(96.69)	535(49.89)	25414(98.30)	
yes	1142(3.31)	552(50.11)	590(1.70)	
NHHR Quartiles				0.03
Q1	5006(18.84)	243(22.94)	4763(18.69)	
Q2	5927(22.10)	231(22.47)	5696(22.09)	
Q3	7336(26.90)	291(25.83)	7045(26.93)	
Q4	8822(32.17)	322(28.77)	8500(32.28)	
CVD				< 0.0001
no	24182(91.53)	0(0.00)	24182(94.68)	
yes	2909(8.47)	1087(100.00)	1822(5.32)	
Hypertension				< 0.0001
no	15594(62.26)	216(23.49)	15378(63.59)	
yes	11497(37.74)	871(76.51)	10626(36.41)	
Mortality status				< 0.0001
Assumed alive	23940(91.42)	658(65.98)	23282(92.29)	
Assumed deceased	3151(8.58)	2722(7.71)	429(34.02)	

NHHR was divided into weighted quartiles: Q1 (< 1.85), Q2 (1.85–2.45), Q3 (2.46–3.10), and Q4 (> 3.10), based on the survey-weighted population distribution

Values are survey-weighted. Continuous variables are presented as mean (SE) and categorical variables as weighted counts (percentage). P-values are from survey-weighted linear regression (continuous) or Rao–Scott *χ*^2^ tests (categorical)

Values are presented as mean (SE) or weighted percentages. Lipid levels are in mmol/L

CHD prevalence was assessed at baseline (cross-sectional), whereas mortality outcomes were evaluated prospectively through follow-up

### Association between NHHR and the prevalence of coronary heart disease (CHD) in adults.

In survey-weighted logistic models, higher NHHR quartiles were associated with lower odds of CHD. Using Q1 as the reference (Table 2), the fully adjusted model (Model 3: age, sex, race/ethnicity, education, BMI, smoking, alcohol use, hypertension, diabetes) showed:

**Table 2:** Association between NHHR quartiles and the prevalence of CHD

NHHR quartiles	Model 1		Model 2		Model 3	
	OR (95% CI)	*p*	OR (95% CI)	*p*	OR (95% CI)	*p*
Q1	ref		ref		ref	
Q2	0.88(0.69–1.13)	0.31	0.76(0.58–0.99)	0.05	0.76(0.58–1.00)	0.05
Q3	0.84(0.65–1.08)	0.17	0.70(0.53–0.92)	0.01	0.70(0.52–0.93)	0.01
Q4	0.73(0.60–0.88)	0.001	0.65(0.52–0.81)	< 0.001	0.60(0.48–0.75)	< 0.0001
NHHR (per 1-SD increase)	0.94(0.89–0.99)	0.02	0.93(0.87–0.99)	0.03	0.92(0.86–0.98)	0.01
*p for trend*	0.002		< 0.0001		< 0.0001	

Model 1 was unadjusted; Model 2 was adjusted for age, sex, ethnicity, education level, and BMI; Model 3 was further adjusted for smoking, drinking status, hypertension, and diabetes

Per-SD corresponds to one standard deviation of NHHR calculated in the survey-weighted population

The analysis of Table 2 demonstrates a consistent inverse association between NHHR quartiles and the prevalence of coronary heart disease (CHD) in adults. In the unadjusted model (Model 1), higher NHHR quartiles were associated with a lower prevalence of CHD, with the strongest effect observed in Q4 (OR = 0.73, 95% CI 0.60–0.88, *P* = 0.001) and a significant P for trend (*P* = 0.002). After adjustment for age, sex, ethnicity, education level, and BMI in Model 2, the associations became more pronounced and statistically significant across all quartiles (Q2: OR = 0.67, 95% CI 0.54–0.84, *P* < 0.001; Q3: OR = 0.50, 95% CI 0.39–0.64, *P* < 0.0001; Q4: OR = 0.37, 95% CI 0.29–0.46, *P* < 0.0001), with a highly significant P for trend (< 0.0001). Further adjustment for smoking, drinking status, hypertension, and diabetes in Model 3 maintained the inverse association, particularly in Q4 (OR = 0.39, 95% CI 0.31–0.50, *P* < 0.0001), indicating that higher NHHR quartiles were consistently associated with a lower prevalence of CHD. The stable P for trend values across all models underscore the robustness of this inverse relationship (Table 2).

To test robustness, a sensitivity analysis was conducted using Q3 as the reference group (see Supplemental Table S1). Compared with Q3, individuals in Q1 showed higher odds of CHD in the adjusted models (Model 2: OR = 1.43, 95% CI 1.09–1.88, *P* = 0.01; Model 3: OR = 1.43, 95% CI 1.08–1.91, *P* = 0.01), suggesting that very low NHHR levels may reflect adverse health conditions or reverse causality. In contrast, Q4 did not show a statistically significant increase in CHD risk compared with Q3 (Model 3: OR = 0.86, 95% CI 0.65–1.13, *P* = 0.28), further supporting the possibility of a nonlinear association.

When NHHR was modeled as a continuous exposure (per 1-SD increase), the odds of CHD decreased (Table 2). In the unadjusted model, each 1-SD higher NHHR was associated with 6% lower odds of CHD (OR = 0.94, 95% CI 0.89–0.99; *P* = 0.02). After adjustment for age, sex, ethnicity, education, and BMI (Model 2), the association was attenuated and not statistically significant (OR = 0.96, 95% CI 0.91–1.02; *P* = 0.23). In the fully adjusted model additionally controlling for smoking, alcohol use, hypertension, and diabetes (Model 3), a 9% lower odds of CHD per 1-SD increase in NHHR was observed (OR = 0.91, 95% CI 0.85–0.97; *P* = 0.01). These continuous-effect estimates are directionally consistent with the quartile analyses, reinforcing a robust inverse association between NHHR and CHD prevalence.

### Establish cox proportional hazard model

Table 3 shows a clear inverse association between NHHR quartiles and both all-cause and cardiovascular disease (CVD) mortality during follow-up. In the unadjusted model (Model 1), higher NHHR levels were linked with a reduced risk of death, particularly in the third and fourth quartiles (all-cause mortality: Q3 HR = 0.79, 95% CI 0.69–0.91, *P* < 0.001; Q4 HR = 0.77, 95% CI 0.68–0.87, *P* < 0.0001; CVD mortality: Q3 HR = 0.72, 95% CI 0.58–0.89, *P* = 0.002; Q4 HR = 0.75, 95% CI 0.61–0.93, *P* = 0.01). After adjusting for demographic and educational factors in Model 2, the protective associations strengthened (all-cause mortality: Q3 HR = 0.69, 95% CI: 0.59–0.80; Q4 HR = 0.62, 95% CI 0.54–0.72; CVD mortality: Q3 HR = 0.61, 95% CI 0.48–0.78; Q4 HR = 0.59, 95% CI 0.46–0.76; all *P* < 0.0001). Further adjustment for BMI, smoking, alcohol use, hypertension, and diabetes in Model 3 continued to demonstrate robust inverse associations (all-cause mortality: Q3 HR = 0.66, 95% CI 0.57–0.77; Q4 HR = 0.61, 95% CI 0.53–0.70; CVD mortality: Q3 HR = 0.58, 95% CI 0.46–0.73; Q4 HR = 0.58, 95% CI 0.45–0.75; all *P* < 0.0001). Consistently significant P for trend values further confirmed the graded protective effect of higher NHHR against mortality.

**Table 3:** Hazard ratios (HRs) for all-cause and cardiovascular mortality across NHHR quartiles

	The quartile of NHHR				
	Q1 (0.205–1.755]	Q2 (1.755–2.396]	Q3 (2.396–3.333]	Q4 (3.333,25.813]	*p* for trend
All-Cause Mortality					
Number of deaths	654	726	825	946	
Model 1 HR (95%CI) *p*	1	0.86(0.77–0.96)0.01	0.78(0.68–0.89) < 0.001	0.77(0.68–0.87) < 0.0001	< 0.0001
Model 2 HR (95%CI) *p*	1	0.82(0.73–0.93) 0.002	0.77(0.67–0.88) < 0.001	0.90(0.80–1.03) 0.13	0.067
Model 3 HR (95%CI) *p*	1	0.82(0.73–0.93)0.001	0.76(0.66–0.87) < 0.0001	0.85(0.75–0.96) 0.01	0.005
CVD Mortality					
Number of deaths	227	227	278	321	
Model 1 HR (95%CI) *p*	1	0.78(0.63–0.96) 0.02	0.74(0.60–0.90) 0.003	0.73(0.59–0.91) 0.005	0.005
Model 2 HR (95%CI) *p*	1	0.77(0.63–0.95) 0.02	0.75(0.62–0.91)0.003	0.97(0.79–1.20) 0.80	0.642
Model 3 HR (95% CI) *p*	1	0.77(0.62–0.94)0.01	0.74(0.62–0.89)0.001	0.93(0.75–1.15) 0.50	0.414

Models as defined in Table 2. Follow-up was from the NHANES examination date through December 31, 2019, using the NCHS linked mortality file

### Nonlinear relationship detection

**Fig. 2:**
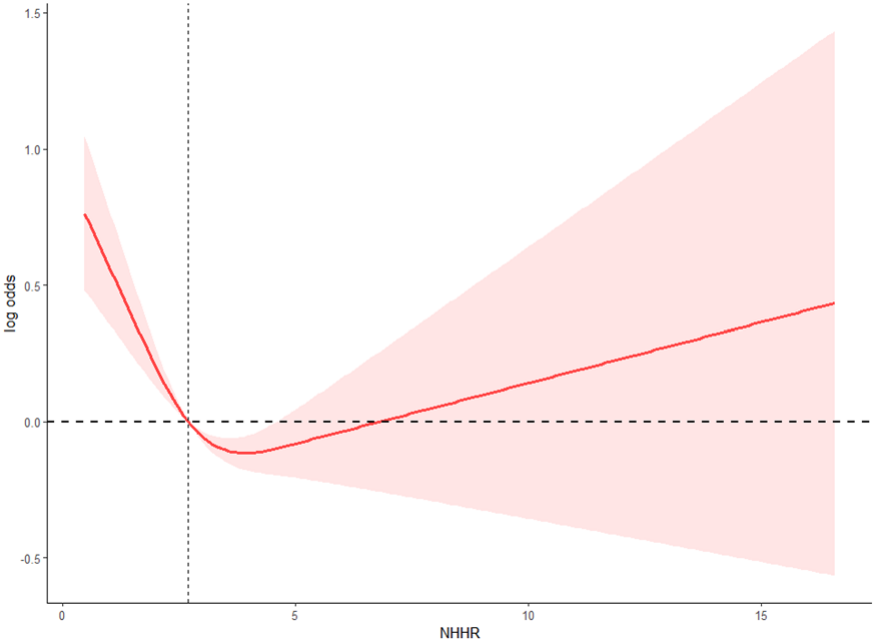
Restricted cubic spline (RCS) curve illustrating the association between NHHR and the prevalence of coronary heart disease (CHD) on the log-odds scale. The model used three knots placed at the 5th, 50th, and 95th percentiles of NHHR and was adjusted for demographic and clinical covariates. The curve shows a statistically significant U-shaped association (*P*-overall < 0.0001; *P*-nonlinear < 0.0001), with the lowest odds of CHD observed at an NHHR value of approximately 3.96. A horizontal reference line at log-odds = 0 (equivalent to OR = 1) and a vertical line at NHHR = 3 were added for interpretability. The shaded area represents the 95% confidence interval

Restricted cubic spline (RCS) analysis, using a predefined three-knot model at the 5th, 50th, and 95th percentiles of NHHR, demonstrated a statistically significant nonlinear association between the non–HDL-C to HDL-C ratio (NHHR) and the prevalence of coronary heart disease (CHD) (*P*-overall < 0.0001; *P*-nonlinear < 0.0001). As shown in Fig. 2, the curve followed a U-shaped pattern: The lowest odds of CHD occurred at an NHHR value of approximately 3.96, with both lower and higher NHHR values associated with increased prevalence. This nonlinear association remained robust after adjusting for demographic and clinical covariates. The reference point was set at the population-weighted median (NHHR ≈ 2.70), and confidence intervals widened toward the extremes, suggesting increased uncertainty in these ranges (Fig. 2).

**Fig. 3:**
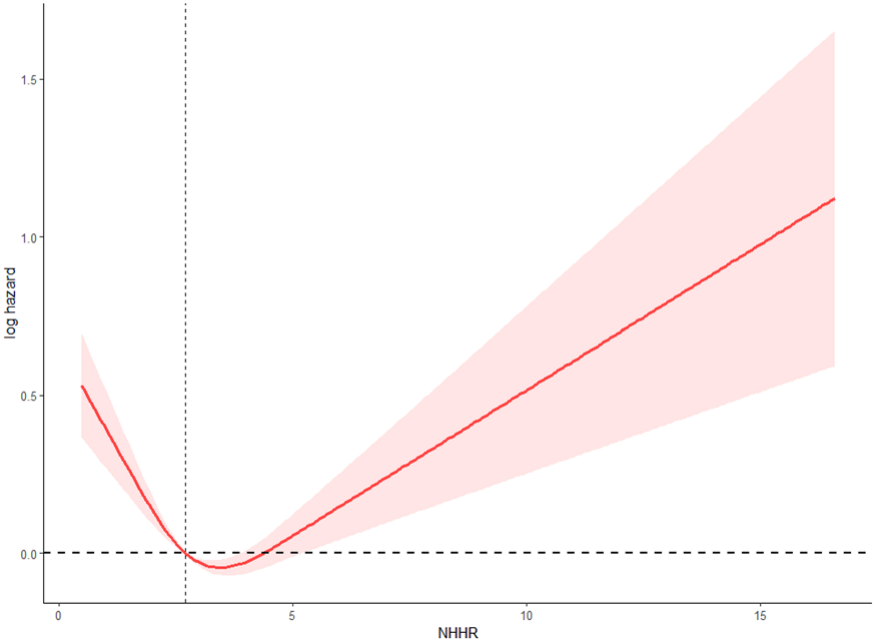
Restricted cubic spline (RCS) plot depicting the relationship between NHHR and all-cause mortality (log-hazard scale). A statistically significant nonlinear association was observed (*P*-overall < 0.0001; *P*-nonlinear < 0.0001) using a three-knot model placed at the 5th, 50th, and 95th percentiles of NHHR. The adjusted spline curve followed a U-shaped pattern, with the lowest estimated log-hazard occurring at an NHHR of approximately 3.47. Horizontal and vertical reference lines were drawn at log-hazard = 0 (HR = 1) and NHHR = 3, respectively. The 95% confidence interval is shown as a shaded band

In prospective analyses, restricted cubic spline (RCS) modeling with a predefined three-knot structure revealed a statistically significant nonlinear association between NHHR and all-cause mortality (*P*-overall < 0.0001; *P*-nonlinear < 0.0001). The adjusted spline curve displayed a U-shaped pattern, with the lowest estimated hazard occurring at an NHHR of approximately 3.47. Both lower and higher NHHR values were associated with increased log-hazards of mortality. Confidence intervals were notably wider at the distribution tails, reflecting lower precision in those regions (Fig. 3).

**Fig. 4:**
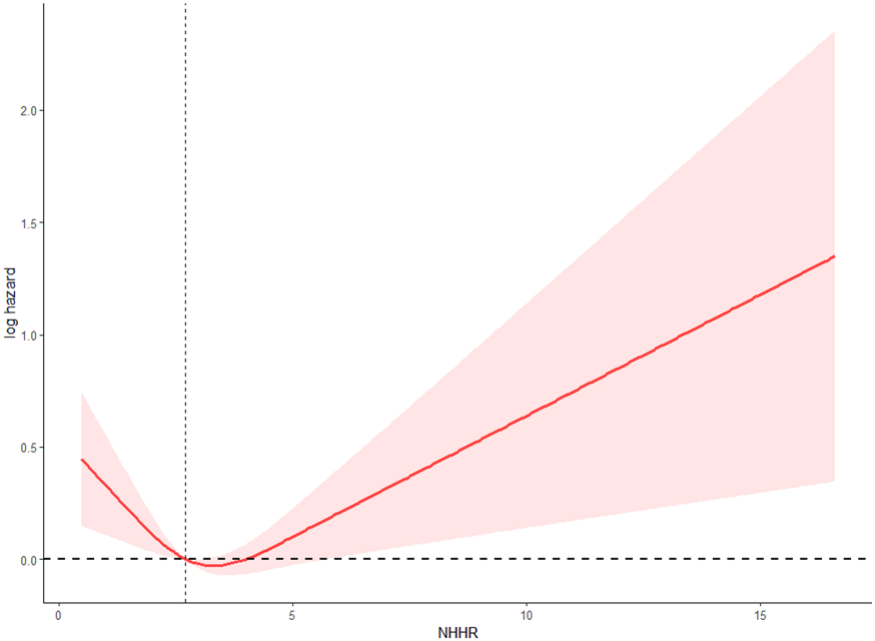
Restricted cubic spline (RCS) curve showing the association between NHHR and cardiovascular mortality (log-hazard scale). The model identified a significant nonlinear trend (*P*-overall = 0.0033; *P*-nonlinear = 0.0008), using the same three-knot structure (5th, 50th, 95th percentiles of NHHR). The adjusted curve displayed a U-shaped relationship, with the lowest hazard observed at an NHHR of approximately 3.31. Dashed reference lines were placed at log-hazard = 0 and NHHR = 3 for clarity. The shaded area reflects the 95% confidence interval

Similarly, RCS modeling indicated a significant nonlinear association between NHHR and cardiovascular mortality, using the same predefined three-knot model (*P*-overall = 0.0033; *P*-nonlinear = 0.0008). The spline curve demonstrated a U-shaped trend, with the nadir observed around an NHHR of 3.31. Increased hazards were noted at both ends of the NHHR range, though with broader confidence intervals at the extremes, indicating greater uncertainty in those regions (Fig. 4).

### Subgroup analysis

Subgroup analyses (Table 4) demonstrated that the inverse association between NHHR and all-cause mortality varied across demographic and clinical characteristics. The protective effect of higher NHHR was more pronounced in males (HR = 0.75, 95% CI 0.67–0.84, *P* < 0.0001) than in females (HR = 1.12, 95% CI 1.00–1.26, *P* = 0.05; P for interaction < 0.0001). A stronger protective effect was also observed among older participants aged ≥ 60 years (HR = 0.45, 95% CI 0.22–0.94, *P* = 0.04; P for interaction = 0.03). Regarding metabolic factors, individuals with diabetes (HR = 0.74, 95% CI 0.64–0.87, *P* < 0.001) or impaired glucose tolerance (IGT) (HR = 0.66, 95% CI 0.53–0.81, *P* < 0.0001) had a significantly reduced risk of mortality, while the association was not evident among those without diabetes. Similarly, overweight participants showed lower mortality risk (HR = 0.81, 95% CI 0.73–0.90, *P* < 0.001; P for interaction < 0.001), whereas no significant effect was detected in obese individuals. Clinical subgroups with pre-existing conditions, including angina (HR = 0.66, 95% CI 0.51–0.84, *P* = 0.001), congestive heart failure (HR = 0.72, 95% CI 0.55–0.93, *P* = 0.01), prior heart attack (HR = 0.75, 95% CI 0.63–0.90, *P* = 0.003), and CVD (HR = 0.77, 95% CI 0.67–0.90, *P* < 0.001), also exhibited stronger protective associations. These findings suggest that the beneficial impact of NHHR on survival is more evident in males, older adults, individuals with glucose metabolism, overweight participants, and those with cardiovascular comorbidities.

**Table 4:** Subgroup analysis of the association between NHHR and all-cause mortality

Variable	HR (95% CI)	*p*	*P* for interaction
Sex			< 0.0001
Female	1.12(1.00–1.26)	0.05	
Male	0.75(0.67–0.84)	< 0.0001	
Ethnicity			0.53
Black	1.00(0.88–1.13)	0.98	
White	0.96(0.87–1.05)	0.36	
Other	0.83(0.66–1.05)	0.13	
Mexican	0.91(0.73–1.15)	0.44	
Education			0.11
Some college or AA degree	0.97(0.82–1.15)	0.70	
College graduate or above	0.96(0.79–1.17)	0.69	
High school graduate/GED or equivalent	0.79(0.68–0.93)	0.004	
9-11th grade (Includes 12th grade with no diploma)	0.74(0.64–0.86)	< 0.001	
Less than 9th grade	0.86(0.70–1.05)	0.15	
Diabetes			0.01
No	0.95(0.84–1.08)	0.44	
Yes	0.74(0.64–0.87)	< 0.001	
IFG	0.79(0.57–1.11)	0.16	
IGT	0.66(0.55–0.81)	< 0.0001	
Alcohol user			0.13
Former	0.84(0.75–0.93)	0.002	
Mild	0.81(0.70–0.94)	0.01	
Never	0.90(0.79–1.03)	0.14	
Moderate	0.87(0.65–1.15)	0.32	
Heavy	1.16(0.84–1.63)	0.37	
Smoke			0.12
Never	0.99(0.87–1.12)	0.83	
Former	0.81(0.71–0.93)	0.003	
Now	0.94(0.78–1.13)	0.49	
Age			0.03
< 60	0.93(0.86–1.01)	0.09	
≥ 60	0.45(0.22–0.94)	0.04	
BMI			< 0.001
Normal weight	1.15(1.02–1.31)	0.03	
Overweight	0.81(0.73–0.90)	< 0.001	
Obesity	0.88(0.76–1.01)	0.07	
Stroke			0.41
No	0.93(0.85–1.02)	0.14	
Yes	0.84(0.66–1.07)	0.16	
Angina			0.003
No	0.95(0.87–1.03)	0.22	
Yes	0.66(0.51–0.84)	0.001	
Congestive heart failure			0.05
Yes	0.72(0.55–0.93)	0.01	
No	0.95(0.87–1.04)	0.26	
Heart attack			0.03
No	0.95(0.87–1.05)	0.33	
Yes	0.75(0.63–0.90)	0.003	
CVD			0.01
Yes	0.77(0.67–0.90)	< 0.001	
No	1.00(0.89–1.12)	0.94	
Hypertension			0.03
Yes	0.81(0.72–0.90)	< 0.001	
No	0.97(0.83–1.12)	0.56	
Coronary heart disease			0.35
No	0.95(0.87–1.04)	0.29	
Yes	0.85(0.68–1.06)	0.14	

As shown in Table 5, subgroup analyses revealed that the protective association between higher NHHR and cardiovascular mortality was more evident in males (HR = 0.70, 95% CI 0.60–0.83, *P* < 0.0001) than in females (HR = 1.12, 95% CI 0.96–1.30, *P* = 0.14; P for interaction < 0.0001). No significant differences were observed across ethnic groups, but individuals with a high school education showed significant reductions in risk (HR = 0.73, 95% CI 0.62–0.86, *P* < 0.001). Participants with impaired glucose regulation, including IFG (HR = 0.60, 95% CI 0.41–0.87, *P* = 0.01) and IGT (HR = 0.59, 95% CI 0.45–0.78, *P* < 0.001), also demonstrated stronger protective effects. Former and mild alcohol users showed significant associations, while heavy drinkers did not. Stratified by BMI, significant reductions in mortality were observed among overweight (HR = 0.75, 95% CI 0.64–0.89, *P* < 0.001) and obese individuals (HR = 0.83, 95% CI 0.70–0.96, *P* = 0.02). Clinically, patients with angina (HR = 0.61, 95% CI 0.44–0.84, *P* = 0.003), congestive heart failure (HR = 0.70, 95% CI 0.53–0.91, *P* = 0.01), and hypertension (HR = 0.76, 95% CI 0.67–0.87, *P* < 0.0001) benefited significantly from higher NHHR. These findings highlight that the inverse association between NHHR and cardiovascular mortality is particularly pronounced among males, individuals with impaired glucose regulation, overweight/obese participants, and those with cardiovascular comorbidities.

**Table 5:** Subgroup analysis of the association between NHHR and cardiovascular mortality

Variable	HR (95% CI)	*p*	*p* for interaction
Sex			< 0.0001
Female	1.12(0.96–1.30)	0.14	
Male	0.70(0.60–0.83)	< 0.0001	
Ethnicity			0.35
Black	1.01(0.86–1.20)	0.89	
White	0.91(0.81–1.02)	0.12	
Other	0.76(0.50–1.14)	0.18	
Mexican	1.14(0.79–1.63)	0.50	
Education			0.57
Some college or AA degree	0.89(0.69–1.13)	0.32	
College graduate or above	0.94(0.70–1.25)	0.66	
High school graduate/GED or equivalent	0.73(0.62–0.86)	< 0.001	
9–11th grade (Includes 12th grade with no diploma)	0.87(0.61–1.19)	0.37	
Less than 9th grade	0.79(0.61–1.01)	0.06	
Diabetes			0.02
No	0.93(0.80–1.09)	0.39	
Yes	0.73(0.61–0.90)	0.002	
IFG	0.60(0.41–0.87)	0.01	
IGT	0.59(0.45–0.78)	< 0.001	
Alcohol user			0.5
Former	0.79(0.66–0.94)	0.01	
Mild	0.84(0.70–0.99)	0.04	
Never	0.93(0.74–1.16)	0.52	
Moderate	0.72(0.52–0.98)	0.04	
Heavy	1.06(0.66–1.70)	0.80	
Smoke			0.72
Never	0.92(0.79–1.06)	0.25	
Former	0.84(0.68–1.03)	0.10	
Now	0.96(0.70–1.30)	0.79	
Age			0.23
< 60	0.90(0.81–1.00)	0.04	
≥ 60	0.47(0.14–1.58)	0.19	
BMI			< 0.001
Normal weight	1.17(1.00–1.38)	0.06	
Overweight	0.75(0.64–0.89)	< 0.001	
Obesity	0.83(0.70–0.96)	0.02	
Stroke			0.81
No	0.89(0.79–1.00)	0.04	
Yes	0.93(0.63–1.38)	0.72	
Angina			0.02
No	0.92(0.83–1.03)	0.16	
Yes	0.61(0.44–0.84)	0.003	
Congestive heart failure			0.07
Yes	0.70(0.53–0.91)	0.01	
No	0.92 (0.82–1.03)	0.15	
Heart attack			0.18
No	0.92(0.82–1.04)	0.18	
Yes	0.76(0.58–0.98)	0.03	
CVD			0.2
Yes	0.79(0.66–0.96)	0.02	
No	0.95(0.81–1.11)	0.49	
Hypertension			0.08
Yes	0.76(0.67–0.87)	< 0.0001	
No	0.99(0.78–1.26)	0.93	
Coronary heart disease			0.85
No	0.91(0.81–1.03)	0.13	
Yes	0.89(0.66–1.19)	0.41	

HRs are from survey-weighted Cox models. Interaction terms were tested by adding the cross-product of NHHR (continuous) and the subgroup variable

## Discussion

We analyzed nationally representative NHANES data (2005–2016) to examine the association between the non-HDL to HDL cholesterol ratio (NHHR) and both CHD prevalence and mortality. While several recent studies [7, 11, 12] using NHANES or other cohort data have evaluated NHHR in relation to cardiovascular outcomes, our study contributes additional insight by examining both cross-sectional CHD prevalence and prospective mortality within the same nationally representative cohort. By integrating quartile-based, continuous, and spline models, and exploring subgroup variations, we provide a more nuanced understanding of potential nonlinearity and effect modification in a general adult U.S. population.

We observed a U-shaped association between NHHR and both CHD prevalence and mortality. While higher NHHR was associated with lower CHD odds at baseline, both very low and very high values were linked to increased mortality during follow-up. In other words, higher NHHR was associated with lower odds of existing CHD in our dataset, even as extremely high or low NHHR values were linked to higher subsequent mortality risk. This pattern may reflect different biological processes at the extremes, since a high NHHR (indicating high non–HDL-C and low HDL-C) is conventionally considered adverse. We have therefore distinguished the cross-sectional nature of the CHD result from the longitudinal nature of the mortality result to avoid any misinterpretation of “predictive” value in the cross-sectional context. Several potential biological mechanisms could explain this counterintuitive U-shaped relationship. At very low NHHR levels, the ratio is typically low because HDL-C is disproportionately high relative to non–HDL-C. Although higher HDL-C is usually viewed as cardioprotective, extremely high HDL-C concentrations may signify dysfunctional HDL particles. Such HDL can have impaired reverse cholesterol transport and even pro-inflammatory effects, or may be a marker of underlying chronic illness or malnutrition; these conditions have all been associated with increased mortality risk in other studies [4]. Conversely, at the opposite extreme, a very high NHHR reflects elevated non–HDL-C together with low HDL-C, which is the classic atherogenic dyslipidemia associated with heightened cardiovascular risk. Thus, intermediate NHHR values (we observed a nadir of risk around NHHR ≈3) might represent an optimal balance point—HDL-C is present at functional levels and non–HDL-C is not excessively elevated. This explanation for the “U-curve” aligns with the idea that both extremes of the ratio capture distinct high-risk states (one related to possible dysfunctional HDL and the other to markedly atherogenic lipid profiles).

Despite these hypotheses, we caution that the observed U-shaped pattern could partly reflect confounding factors or reverse causation. For example, individuals with higher cardiovascular risk may be more likely to receive lipid-lowering therapies (e.g., statins) that lower non–HDL-C (and thus NHHR) while also reducing CHD events. This would result in some high-risk individuals showing lower NHHR values at baseline, potentially biasing the association toward an inverse relationship. Additionally, survivors of CHD or other illnesses might adopt aggressive treatments and lifestyle modifications that lower their NHHR after diagnosis (for instance, through intensive statin use and diet changes), making them overrepresented in the low-NHHR group. To explore these issues, we considered the potential confounding effect of lipid-lowering medications. However, due to inconsistent availability of medication data across NHANES cycles, a formal sensitivity analysis excluding statin users could not be conducted. This limitation may partially contribute to the paradoxical associations observed. This suggests that while medication use and survivor bias may contribute to the paradoxical findings, they do not entirely explain the association. This paradoxical inverse association between NHHR and CHD may be partly explained by treatment-related reverse causality. Individuals with known CHD are more likely to receive lipid-lowering therapies or implement lifestyle modifications (e.g., dietary changes, increased physical activity), which can lower NHHR at the time of measurement. Thus, a proportion of high-risk individuals may appear in lower NHHR categories. Additionally, survivors with prior CHD may represent a selectively healthier subset who are engaged in secondary prevention. Furthermore, as CHD diagnosis is based on self-report, under-reporting among individuals with high NHHR but no prior diagnosis may contribute to this inverse pattern. These interrelated factors may lead to an apparent protective association that does not reflect true underlying risk.

Our analysis identified a U-shaped association between NHHR and both self-reported CHD prevalence and prospective mortality. Both extremely low and high NHHR values appeared to be associated with elevated risk, with the lowest observed risk occurring at an intermediate NHHR of approximately 3. While the biological explanation for this pattern remains uncertain, one possible mechanism involves the distinct physiological states represented at the extremes. Very low NHHR may be driven by disproportionately high HDL-C levels, which—despite traditionally being considered protective—may reflect dysfunctional HDL particles under certain conditions. Prior studies have associated extremely high HDL-C with impaired reverse cholesterol transport, pro-inflammatory activity, and comorbidities such as chronic illness or malnutrition, all of which could contribute to increased risk [4]. At the opposite end, high NHHR typically reflects elevated non–HDL-C and reduced HDL-C—features characteristic of an atherogenic lipid profile consistent with traditional cardiovascular risk factors. Together, these contrasting mechanisms may contribute to the observed U-shaped curve. However, the observed nadir around NHHR ≈3 should not be interpreted as a clinical threshold. Further validation is required in diverse, clinically adjudicated populations before NHHR can be used for individual-level risk stratification or clinical decision-making. At the lower end of the curve, low NHHR is often driven by very high HDL-C relative to non–HDL-C. While HDL-C has traditionally been viewed as protective, emerging evidence indicates that extremely elevated HDL-C may be dysfunctional—impairing reverse cholesterol transport and acquiring pro-inflammatory properties. Moreover, such lipid profiles may reflect underlying chronic illness, frailty, or catabolic states—particularly in older adults—which are associated with increased mortality. From a statistical perspective, this creates a non-monotonic risk pattern, where both ends of the distribution are linked to vulnerability, thus producing a U-shaped curve rather than a linear association.

Our findings also need to be contextualized relative to previous studies. Interestingly, some earlier reports have shown different patterns. For instance, You et al. (2020) [5] studied a high-risk Chinese hospital cohort undergoing coronary angiography and found a positive linear correlation between NHHR and coronary artery disease-higher NHHR unequivocally corresponded to greater CAD risk. By contrast, our general population analysis revealed a U-shaped association, including an inverse relationship at higher NHHR levels (i.e., individuals with the highest ratios did not have the highest CHD prevalence or mortality risk). This discrepancy likely arises from differences in study design and population: You et al. examined angiographically confirmed disease in a symptomatic, high-risk sample, whereas we relied on self-reported CHD in a broader U.S. population. The use of self-reported CHD in NHANES, as opposed to clinically adjudicated diagnoses used in hospital-based cohorts, presents a potential source of misclassification bias. Self-report relies on participants’ recall of physician diagnoses, which may under-detect subclinical or undiagnosed disease, especially among individuals with limited access to care or lower health literacy. This may result in non-differential misclassification, potentially attenuating observed associations. Additionally, differential misclassification is possible if individuals with certain lipid profiles (e.g., very high NHHR) are more likely to be diagnosed or recall a diagnosis. Compared with studies using angiographically confirmed coronary artery disease (CAD), such as You et al. [5], our reliance on self-reported CHD may contribute to the observed inverse association by underestimating disease prevalence in high-risk subgroups. Future studies with clinically adjudicated endpoints are needed to validate the robustness and directionality of these associations.

The hospital-based cohort had a higher disease burden and more precise outcome assessment, which may produce a more straightforward risk gradient. Conversely, factors like treatment effects and health status variations in the general population can lead to nonlinear patterns. Notably, several other studies in specific populations have reported nonlinear (often U-shaped) associations between NHHR and outcomes. For example, Ma et al. (2025) [7] and Su et al. (2025) [12] each observed U-shaped NHHR–mortality relationships in patients with hypertension, and Yang et al. (2025) [11] documented a similar U-curve in U.S. adults with nonalcoholic fatty liver disease. Likewise, Chang et al. (2022) [9] demonstrated a U-shaped NHHR–mortality curve in septic patients, and Huang et al. (2023) [13] noted a nonlinear association between non-HDL cholesterol and mortality in a general adult cohort. These studies differed in their outcome definitions (clinical adjudication vs. self-report), population characteristics (disease-specific versus general populations), and potential confounders, which could shift the shape or inflection points of the risk curves. Nonetheless, the recurring observation of nonlinear trends across diverse settings reinforces the notion that NHHR captures complex, context-dependent risk dynamics beyond a simple linear effect.

Our subgroup analyses further illuminate how NHHR’s association with outcomes varies by sex. There are well-established sex differences in lipid metabolism that may modulate the interpretation of NHHR. Premenopausal women generally have higher HDL-C and lower non–HDL-C levels than men, largely due to estrogen’s favorable effects on lipoprotein metabolism (i.e., increased HDL-C and decreased LDL-C), whereas androgens exert the opposite influence [14, 15]. These hormonal effects result in more favorable NHHR values in women. Consequently, a given NHHR may indicate a higher atherogenic burden and greater risk in men than in women. Indeed, we observed stronger associations between NHHR and adverse outcomes in men, likely reflecting the absence of estrogen-mediated lipid protection and a less favorable baseline lipid profile. Importantly, metabolic conditions such as diabetes may attenuate these sex-based differences. In diabetic women, the usual HDL advantage is diminished due to both quantitative and functional impairments in HDL [14, 15], which may explain the reduced sex disparity in NHHR-related risk among participants with dysglycemia.

Age also modifies the relationship between NHHR and mortality. In younger adults (< 50 years), elevated non-HDL-C has been shown to carry stronger prognostic value16. In this context, a high NHHR likely reflects early-onset atherogenic exposure with cumulative lifetime risk. In contrast, among older or frail adults, low NHHR may reflect reverse causation—i.e., declining lipid levels due to chronic illness, malnutrition, or systemic inflammation. This phenomenon aligns with the “cholesterol paradox,” wherein low cholesterol levels paradoxically associate with increased mortality risk in vulnerable populations [17, 18]. Prior studies have reported U-shaped associations between lipid parameters and mortality in hypertensive or elderly groups [17, 18]. Our findings are consistent with this pattern, as we observed increased mortality risk at both extremes of the NHHR spectrum in older adults.

Metabolic comorbidities such as diabetes and obesity may further influence NHHR-related risk. Individuals with diabetes frequently exhibit atherogenic dyslipidemia and impaired HDL function, both of which could amplify NHHR’s predictive capacity. In our study, the association between NHHR and all-cause mortality was particularly pronounced in diabetic participants, consistent with prior reports [19]. Similarly, obesity-related chronic inflammation may impair HDL-mediated cholesterol efflux and endothelial protection, thereby increasing cardiovascular vulnerability even at similar NHHR levels [19, 20]. These findings support the notion that NHHR may serve as a more informative risk marker in populations with underlying metabolic dysfunction.

In summary, the prognostic value of NHHR appears to be modified by sex hormones, age-related health status, and the presence of metabolic comorbidities. Such interactions should be considered when interpreting NHHR in diverse clinical contexts. Composite lipid markers like NHHR, the triglyceride/HDL-C ratio, or the apolipoprotein B/A1 ratio may offer added value in risk stratification beyond conventional lipid measures. Nevertheless, given the observational nature of our study, these associations remain exploratory. Further mechanistic and prospective investigations are needed to validate NHHR’s clinical utility across different subgroups.

## Limitations

Several limitations of our analysis should be acknowledged. First, CHD status in NHANES was based on self-report rather than clinically adjudicated diagnoses. Although commonly used in population studies, self-reported data are vulnerable to recall bias and misclassification, which may attenuate associations and introduce subgroup variability. Second, the cross-sectional design of the NHANES CHD data restricts our findings to associations with prevalent disease, precluding inference about causality or incident CHD. Third, NHHR and all covariates were measured at a single time point, limiting our ability to capture longitudinal changes or cumulative exposure, potentially leading to exposure misclassification.

Fourth, statin use data were inconsistently available across survey cycles, preventing complete adjustment for lipid-lowering therapy. As statins reduce non-HDL-C and cardiovascular risk, this limitation may have introduced residual confounding. Although we attempted to assess the role of lipid-lowering therapy, the lack of consistent data on statin use across survey years precluded robust adjustment or exclusion. As a result, residual confounding from medication use remains possible. Fifth, NHANES lacks measures of HDL particle function, systemic inflammation, and nutritional status. These unmeasured factors may help explain elevated risk at low NHHR levels. For instance, high HDL-C could reflect dysfunctional HDL or underlying catabolic states.

Finally, our findings are based on a U.S. adult population and may not generalize to other countries or ethnic groups with differing risk profiles or healthcare systems. Variation in NHANES methods across cycles could also contribute to measurement variability. These limitations emphasize the need for replication in longitudinal studies with adjudicated outcomes, comprehensive treatment data, and mechanistic biomarkers to assess the predictive value of NHHR and clarify underlying pathways. While prior NHANES-based studies have investigated NHHR in specific clinical subgroups or single outcomes, our study adds complementary evidence by jointly analyzing CHD prevalence and long-term mortality using harmonized analytic strategies in a nationally representative dataset.

## Conclusions

In summary, using a nationally representative U.S. adult cohort, we observed that NHHR was inversely associated with CHD prevalence in cross-sectional analysis and exhibited a U-shaped association with all-cause and cardiovascular mortality in longitudinal follow-up. These findings, consistent with and extending prior research, suggest that NHHR may reflect complex, context-dependent cardiovascular risk dynamics. Given the observational nature of our analysis and the use of self-reported CHD data, our results should be interpreted cautiously and viewed as exploratory. Further studies—particularly those using clinically adjudicated outcomes and longitudinal lipid measurements—are needed to clarify whether and how NHHR might inform individualized cardiovascular risk assessment.

## Supplementary Information

Supplementary Material 1

## Data Availability

The data that support the findings of this study are available from the National Health and Nutrition Examination Survey (NHANES), which is publicly accessible through the Centers for Disease Control and Prevention (CDC). These data can be accessed at https://www.cdc.gov/nchs/nhanes/. All data generated or analyzed during this study are included in this published article.
