# Supplementary material for: Non–HDL to HDL cholesterol ratio and coronary outcomes in U.S. adults: a cross-sectional and prospective NHANES analysis
Source: Eur J Med Res. 2026 Jan 30;31:350. doi: 10.1186/s40001-026-03977-x (PMC12930730; doi:10.1186/s40001-026-03977-x)
Supplement: Supplementary file 1 — Supplementary Material 1 [file 40001_2026_3977_MOESM1_ESM.docx]

**Supplemental**

**Table S1. Sensitivity analysis of NHHR quartiles and CHD prevalence using Q3 as the reference.**

| **NHHR Quartiles** | **Model 1** | | **Model 2** | | **Model 3** | |
| --- | --- | --- | --- | --- | --- | --- |
|  | OR (95% CI) | *P* | OR (95%CI) | *P* | OR (95%CI) | *P* |
| Q3 | ref | | ref | | ref | |
| Q1 | 1.19(0.93-1.53) | 0.17 | 0.01 | 1.43(1.09-1.88) | 1.43(1.08-1.91) | 0.01 |
| Q2 | 1.05(0.85-1.29) | 0.64 | 0.48 | 1.08(0.86-1.36) | 1.09(0.87-1.38) | 0.45 |
| Q4 | 0.87(0.68-1.10) | 0.24 | 0.56 | 0.93(0.71-1.20) | 0.86(0.65-1.13) | 0.28 |
| P for trend | 0.11 | | 0.26 | | 0.11 | |

**Noted**: This analysis used Q3 as the reference to assess robustness to the choice of referent.
